# Drug-induced kidney disease: a study of the Japan Renal Biopsy Registry from 2007 to 2015

**DOI:** 10.1007/s10157-015-1201-4

**Published:** 2015-11-21

**Authors:** Hitoshi Yokoyama, Ichie Narita, Hitoshi Sugiyama, Michio Nagata, Hiroshi Sato, Yoshihiko Ueda, Seiichi Matsuo

**Affiliations:** 1 Department of Nephrology, Kanazawa Medical University School of Medicine, 1-1 Daigaku, Uchinada, Ishikawa 920-0293 Japan; 2 Division of Clinical Nephrology and Rheumatology, Niigata University Graduate School of Medical and Dental Sciences, Niigata, Japan; 3 Department of Medicine and Clinical Science, Okayama University Graduate School of Medicine, Dentistry, and Pharmaceutical Sciences, Okayama, Japan; 4 Department of Pathology, Faculty of Medicine, University of Tsukuba, Ibaragi, Japan; 5 Clinical Pharmacology and Therapeutics, Tohoku University Graduate School of Pharmaceutical Sciences, Sendai, Japan; 6 Department of Pathology, Dokkyo Medical University Koshigaya Hospital, Saitama, Japan; 7 Department of Nephrology, Nagoya University Graduate School of Medicine, Nagoya, Japan

**Keywords:** Drug, Kidney injury, Japanese, Nephrotic syndrome, Tubulointerstitial nephritis

## Abstract

**Introduction:**

The Japan Renal Biopsy Registry (J-RBR) was started in 2007 by the Committee for the Standardization of Renal Pathological Diagnosis and the Committee for the Kidney Disease Registry of the Japanese Society of Nephrology. The purpose of this report is to clarify drug-induced kidney disease (DIKD) of renal biopsied cases in Japan.

**Subjects and methods:**

We analyzed the data of 26,535 cases that were registered in the J-RBR from 2007 to 2015.

**Results:**

Based on clinical and pathological diagnoses, 328 cases (176 males and 152 females) of renal biopsy-proven DIKD were registered in the J-RBR from 2007 to 2015 (1.24 % of all cases). The frequency of DIKD increased with age. The number of cases peaked in the 6th–8th decade in all pathological categories, except for the number of chronic tubulointerstitial lesions (CTIL), which peaked in the 4th–5th decade. Overall, the frequency of DIKD was 3 times higher in the 7th decade than in the 2nd decade (1.86 vs. 0.62 %). The main clinical diagnoses were DIKD in 150 cases (45.7 %), nephrotic syndrome in 66 cases (20.1 %), chronic nephritic syndrome in 55 cases (16.8 %), and rapidly progressive glomerulonephritis in 30 cases (9.1 %). DIKD was registered as a secondary diagnosis in 136 cases (41.5 %). The pathological findings of these cases were glomerular lesions in 105 cases (32.0 %), acute tubulointerstitial lesions (ATIL) in 87 cases (26.5 %), CTIL in 72 cases (22.0 %), and sclerotic glomerular lesions and/or nephrosclerosis in 18 cases (5.5 %). ATIL and CTIL were mainly found in cases in which DIKD was diagnosed on the basis of the patient’s clinical findings. In addition, nephrotic syndrome-related membranous nephropathy (MN) was the major cause of renal damage in 59.4 % of the cases involving glomerular injuries. According to the CGA risk classification, high-risk (red zone) cases accounted for 56.1 % of all cases of DIKD and 75.9, 64.9, and 33.3 % of the cases involving ATIL, CTIL, and glomerular injuries, respectively. The causative drugs were identified in 102 cases, including bucillamine in 38 cases of MN, gemcitabine in 3 cases of thrombotic microangiopathy, and other anticancer drugs in 14 cases (anti-vascular endothelial growth factor drugs in 3 cases and propyl thiouracil in 3 cases of anti-neutrophil cytoplasmic antibody-related nephritis).

**Conclusion:**

Our analysis of the J-RBR revealed that DIKD mainly affects elderly people in Japan. ATIL or CTIL were found in approximately half of the biopsied cases of DIKD, and one-third involved glomerular lesions, mainly MN or clinical nephrotic syndrome.

**Electronic supplementary material:**

The online version of this article (doi:10.1007/s10157-015-1201-4) contains supplementary material, which is available to authorized users.

## Introduction

The Japanese Society of Nephrology (JSN) established the Japan Renal Biopsy Registry (J-RBR) in 2007, and in 2011 it conducted the first analysis of the registry, which examined the data for 2007 and 2008 [1]. In 2009, the JSN started the Japan Kidney Disease Registry (J-KDR) to record clinically diagnosed cases of kidney disease. The clinical training hospitals of the JSN were requested to add data to this nationwide registry. Based on these data, annual reports [1, 2], epidemiological and cross-sectional studies of membranous nephropathy (MN) [3], elderly patients with renal disease [4], diabetic nephropathy [5], and renal disease combined with obesity [6], and a retrospective study of the outcomes of elderly patients with nephrotic syndrome [7] have been reported, which have helped to clarify the epidemiology of biopsied and unbiopsied renal disease in Japan.

Drug-induced kidney disease (DIKD) accounts for 19–26 % of cases of acute kidney injury (AKI) among hospitalized patients [8]. Recently, Mehta et al. have developed consensus definitions for DIKD based on the patient’s clinical presentation, which take into account the wide spectrum of the condition and the need to balance practicality with reliability. They proposed 4 phenotypes of DIKD: AKI, glomerular disorders, tubular dysfunction, and nephrolithiasis [9]; however, there are no biopsy-proven nationwide epidemiological data about DIKD, even in Japan. Thus, we should develop methods of identifying DIKD and produce preventive plans to protect patients from developing the condition.

In this report, the data about renal biopsy-proven DIKD that were registered in the J-RBR between July 2007 and June 2015 are summarized, and the frequency of the condition is analyzed according to clinicopathological diagnosis and age.

## Subjects and methods

### Registry system and patients

This report includes data for the patients that were prospectively registered in the J-RBR between July 2007 and June 2015. The patients’ data, including their age, gender, laboratory findings, and clinical and pathological diagnoses were recorded at each institution and registered on the webpage of the J-RBR via the Internet Data and Information Center for Medical Research (INDICE) system of the University Hospital Medical Information Network (UMIN), as described previously [1]. The ethics committee of the JSN approved the study protocol, as did the local committees of the participating centers and their affiliated hospitals. Written informed consent was obtained from the patients at the time of biopsy or at the time they were registered to participate in the study. The J-RBR is registered in the UMIN Clinical Trials Registry (registered number: UMIN000000618).

### Clinical or renal histopathological diagnosis and laboratory data

The clinical diagnosis, the histological diagnosis based on the pathogenesis of the disease, and the histological diagnosis based on a histopathological examination were recorded for each case included in the J-RBR, as described previously [1]. Each diagnosis was based on the patients’ clinical symptoms and renal histopathology, as described previously [10]. IgA nephropathy (Berger’s disease) was differentiated from primary glomerular disease on the basis of the glomerular alterations it causes, as described in the classification of glomerular diseases produced by the World Health Organization [10].

Clinical data, including urinalysis results and daily proteinuria, serum creatinine (Cr), total protein, albumin, and total cholesterol levels were recorded in all cases, while recording data regarding systolic and diastolic blood pressure, the use of anti-hypertensive agents, hemoglobin A1c values, and the presence/absence of diabetes mellitus were considered to be optional. The estimated glomerular filtration rate (eGFR) was calculated based on the patients’ serum Cr levels, as described previously [11].

### Analyses of the frequency of DIKD according to age and risk category (the heat map method)

We analyzed the frequency of DIKD according to age. In addition, the frequency of high-risk (red zone) cases was assessed in various arbitrary pathological categories, such as glomerular lesions, acute tubulointerstitial lesions (ATIL), chronic tubulointerstitial lesions (CTIL), and sclerotic glomerular lesions and/or nephrosclerosis (sclerotic lesions), based on the CGA risk classification for chronic kidney disease (the heat map method) [12].

### Statistical analyses

Data are expressed as the mean ± SD for continuous parametric data, the median and interquartile range for continuous non-parametric data, and as frequencies for categorical data.

Comparisons of categorical variables among groups of different indications or diagnoses were performed using Fisher’s exact test. Continuous variables were compared using ANOVA for parametric data and the Kruskal–Wallis test for non-parametric data. All statistical analyses were performed using SPSS version 18.0 (SPSS, Tokyo, Japan).

## Results

### Baseline characteristics of the patients included in the J-RBR and the DIKD patients

**Fig. 1:**
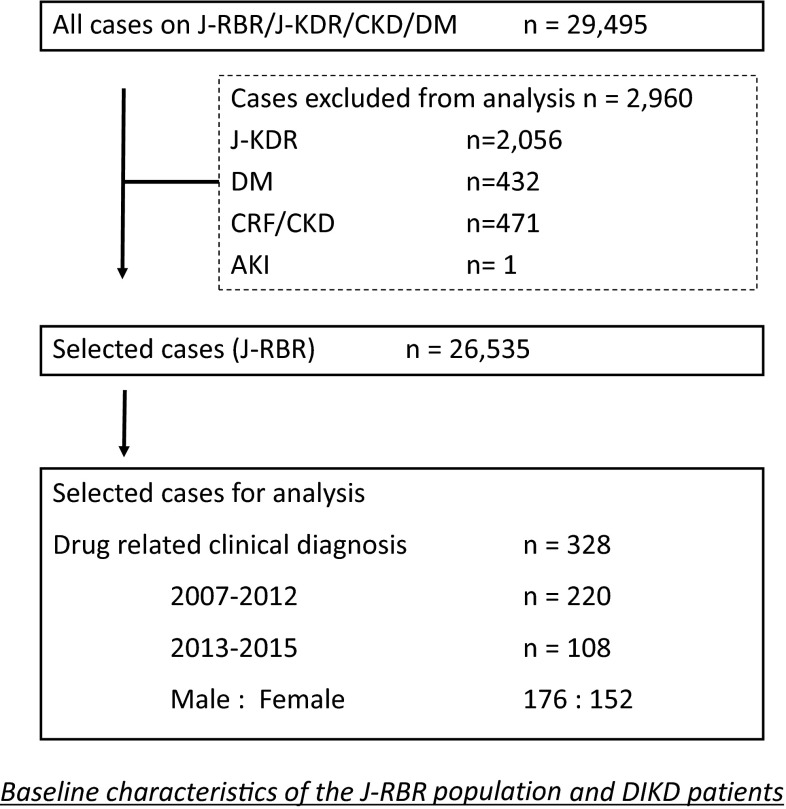
Baseline characteristics of the J-RBR population and the drug-induced kidney disease patients. From among the 26,535 cases that were registered in the J-RBR between 2007 and 2015, 328 cases (176 males and 152 females) of renal biopsy-proven DIKD were extracted based on their clinical and/or pathological diagnoses

Among the cases included in the registry from July 2007 to June 2015, the numbers of cases in which renal biopsies were and were not performed are shown in Fig. 1. From this cohort, 328 cases (176 males and 152 females) of renal biopsy-proven DIKD were extracted based on their clinical and/or pathological diagnoses (1.24 % of the 26,535 cases registered in the J-RBR from 2007 to 2015).

There was no significant difference in the frequency of DIKD between the first period (July 2007–June 2012) and the second period (July 2012–June 2015) (220 cases out of 17,297 cases, 1.27 % vs. 108 cases out of 9238 cases, 1.17 %; not significant).

### The frequency of clinical and pathological diagnoses in DIKD

The main clinical diagnoses were DIKD in 150 cases (45.7 %), nephrotic syndrome in 66 cases (20.1 %), chronic nephritic syndrome in 55 cases (16.8 %), and rapidly progressive nephritic syndrome in 30 cases (9.1 %). DIKD was registered as a secondary diagnosis in 136 cases (41.5 %); thus, 286 cases (87.2 %) were diagnosed as DIKD based on their clinical symptoms (Table 1). Another 36 cases (12.8 %) were diagnosed as DIKD based on their pathological findings.

**Table 1:** Clinical diagnoses of the cases of drug-induced kidney disease in the J-RBR (2007–2015)

Clinical diagnosis	Cases	%
DIKD^a^	150	45.7
Chronic nephritic syndrome + DIKD^a^	46	14.0
Nephrotic syndrome + DIKD^a^	45	13.7
RPGN^b^ + DIKD^a^	29	8.8
Nephrotic syndrome	17	5.2
Acute nephritic syndrome + DIKD^a^	11	3.4
Acute kidney injury	5	1.5
Chronic nephritic syndrome	5	1.5
Recurrent hematuria + DIKD^a^	4	1.2
Chronic nephritic syndrome + others	4	1.2
Others	3	0.9
Nephrotic syndrome + others	2	0.6
Nephrotic syndrome + collagen disease/vasculitis	2	0.6
HUS/TTP^c^	1	0.3
Acute nephritic syndrome + acute kidney injury	1	0.3
Acute kidney injury + DIKD^a^	1	0.3
RPGN^b^	1	0.3
Collagen disease/vasculitis	1	0.3
Total	328	100

Based on their clinical symptoms, 136 patients (41.5 %) were given a secondary diagnosis of DIKD, and a total of 286 patients (87.2 %) were diagnosed with DIKD

J-RBR, Japan Renal Biopsy Registry; ^a^DIKD, drug-induced kidney disease; ^b^RPGN, rapidly progressive glomerulonephritis; ^c^HUS/TTP, hemolytic uremic syndrome/thrombotic thrombocytopenic purpura

The pathological findings of these cases included glomerular lesions in 105 cases (32.0 %), ATIL in 87 cases (26.5 %), CTIL in 72 cases (22.0 %), and sclerotic glomerular lesions and/or nephrosclerosis in 18 cases (5.5 %) (Table 2, Supplemental Table 1). ATIL and CTIL were most commonly associated with a clinical diagnosis of DIKD. In addition, nephrotic syndrome-related MN was the major cause of glomerular lesions in 59.4 % of the cases involving glomerular lesions (Table 2).

**Table 2:** Pathological categories of the cases of drug-induced kidney disease in the J-RBR (2007–2015)

Pathological diagnosis	Cases	%
*Acute kidney injuries*	87	26.5
Acute tubulointerstitial nephritis	76	23.2
Acute tubular necrosis	11	3.4
*Chronic tubulointerstitial lesions*	72	22.0
*Glomerular disorders*	105	32.0
Membranous nephropathy	63	19.2
Minor glomerular abnormalities	14	4.3
Mesangial proliferative glomerulonephritis	12	3.7
Crescentic glomerulonephritis	8	2.4
Membranoproliferative glomerulonephritis Type I or III	3	0.9
Focal segmental glomerulosclerosis	3	0.9
Endocapillary proliferative glomerulonephritis	2	0.6
*Sclerotic lesions*	18	5.5
Nephrosclerosis	14	4.3
Sclerosing glomerulonephritis	4	1.2
*Others*	45	13.7
Transplanted kidney	1	0.3
*Total*	328	100

J-RBR, Japan Renal Biopsy Registry

The frequencies of the 3 major pathological categories did not differ significantly between the first (July 2007–June 2012) and second periods (July 2012–June 2015) (Supplemental Table 1).

### The numbers of cases and frequency of DIKD according to age and pathological category

**Fig. 2:**
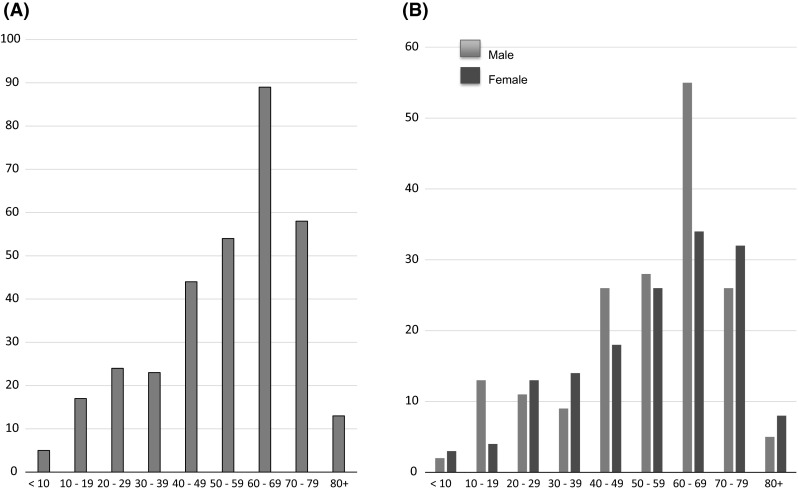
Number of cases of drug-induced kidney disease in the J-RBR. **a** The number of cases of drug-induced kidney disease increased with age, peaking in the 7th decade*. **b** The number of cases peaked in the 6th–8th decade in both genders, and the males exhibited an especially marked peak in the 7th decade (*data for each decade are shown in Supplemental Table 2)

The total number of cases of DIKD is shown in Fig. 2 and Supplemental Table 2, and the frequency of DIKD increased with age until the 7th decade (Fig. 2a). The number of cases of DIKD peaked in the 6th–8th decade in both genders, and in males a particularly marked peak was seen in the 7th decade (Fig. 2b).

**Fig. 3:**
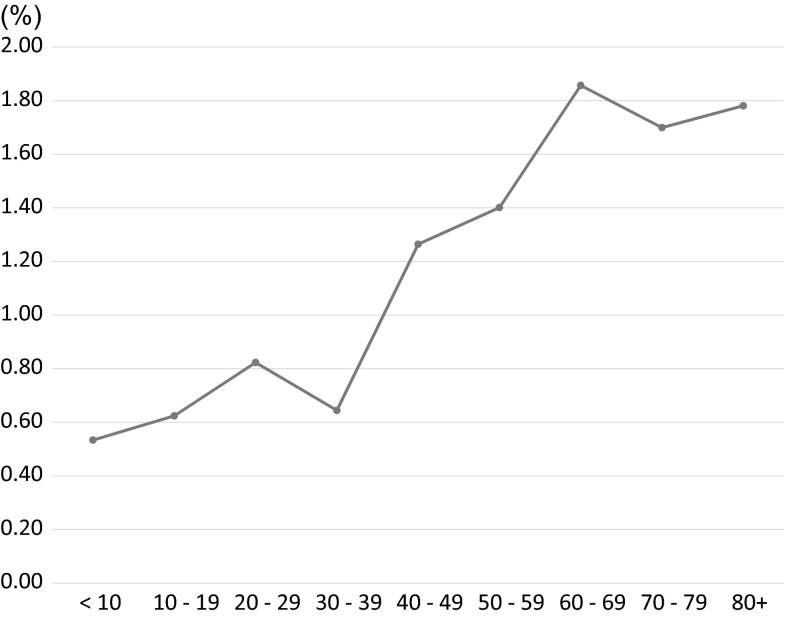
The frequency of drug-induced kidney disease among renal biopsied cases according to age. The frequency of drug-induced kidney disease increased with age and peaked in the 7th decade*. The frequency of drug-induced kidney disease was elderly 3 times higher in the 7th decade than in the 2nd decade (1.86 vs. 0.62 %) (*data for each decade are shown in Supplemental Table 3)

**Fig. 4:**
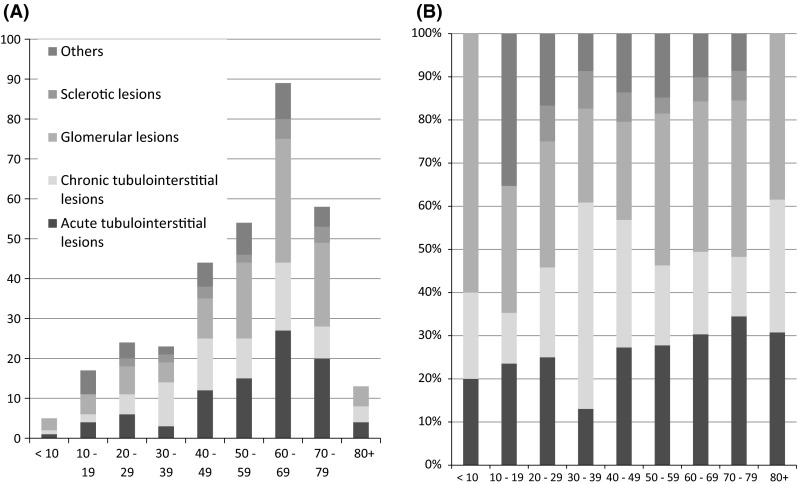
The frequency of each pathological category of drug-induced kidney disease according to age. **a** Total number of cases in each pathological category, **b** the frequency of each pathological category in each decade. The number of cases peaked in the 6th–8th decade in all pathological categories, except for the number of cases involving chronic tubulointerstitial lesions, which peaked in the 4th–5th decade

As for the frequency of DIKD among the renal biopsied cases in each decade, the frequency of DIKD was 3 times higher in the 7th decade than in the 2nd decade (1.86 vs. 0.62 %) (Fig. 3, Supplemental Table 3). The number of cases of DIKD also peaked in the 6th–8th decade in all pathological categories, except for in the cases involving CTIL, in which it peaked in the 4th–5th decade (Fig. 4a, b, Supplemental Fig. 2).

### Baseline clinical and laboratory characteristics of the DIKD patients

The patients’ urinary findings are shown in Table 3. In dipstick tests, 104 cases (31.7 %) were classified as (−) or (±), and 83 cases (25.3 %) were classified as ≥3+. Similarly, 77 cases (32.5 %) and 58 cases (24.2 %) involved patients that exhibited daily proteinuria values of <0.3 g/day (*n* = 237) or urinary protein/creatinine ratios (UPCR) of <0.3 g/gCr (during spot urine tests) (*n* = 240). In addition, 86 cases (36.3 %) and 128 cases (51.7 %) involved patients that demonstrated daily proteinuria values of ≥0.3 g/day and UPCR of ≥0.3 g/gCr (in spot urine tests), respectively (Table 3). As for the results obtained in each pathological category, in the ATIL and CTIL categories 30 cases (32.6 %) and 35 cases (48.6 %), respectively, were classified as (−) or (±) during dipstick tests (Table 4).

**Table 3:** Urinalysis results of all cases of drug-induced kidney disease

Urinary protein (dipstick test)	Cases	%
(−)	67	20.4
(±)	37	11.3
1+	75	22.9
2+	66	20.1
3+	55	16.8
4+	28	8.5
Total	328	100.0

Daily proteinuria levels and urinary protein levels according to spot urine tests					
g/day	Cases	%	g/gCr	Cases	%
<0.30	77	32.5	<0.30	58	24.2
0.30–0.49	22	9.3	0.30–0.49	19	7.9
0.50–0.99	52	21.9	0.50–0.99	39	16.3
1.00–3.49	45	19.0	1.00–3.49	64	26.7
3.50+	41	17.3	3.50+	60	25.0
Total	237	100.0	Total	240	100.0

Hematuria (occult blood grade and red blood cell grade of urinary sediment)					
OB	Cases	%	/hpf	Cases	%
(−)	144	43.9	(−)	91	27.7
(±)	51	15.5	<5	144	43.9
1+	41	12.5	5–10	28	8.5
2+	46	14.0	<10–30	26	7.9
3+	46	14.0	Many	39	11.9
Total	328	100.0	Total	328	100.0

OB, occult blood levels according to the dipstick test; hpf, high-powered field

**Table 4:** Demographic characteristics of the patients with the 3 major pathological subtypes of drug-induced kidney disease

Category	ATIL (92 cases)		CTIL (72 cases)		Glomerular lesions (106 cases)		*p* value*
	Mean	SD	Mean	SD	Mean	SD	
Age	56.9	18.49	52.9	18.59	55.79	19.6	0.346
Height (cm)	159.0	11.2	159.6	11.4	156.1	12.3	0.020
Weight (kg)	57.4	14.4	55.0	12.1	55.6	12.5	0.176
BMI	22.5	4.4	21.5	3.8	22.6	3.7	0.390
Systolic BP (mmHg)	126.9	22.5	121.5	17.5	126.3	19.3	0.314
Diastolic BP (mmHg)	73.8	14.4	73.2	14.0	75.8	11.9	0.576
Mean BP (mmHg)	91.5	16.0	89.3	14.0	92.6	13.3	0.455
Daily proteinuria (g)	0.73	1.00	0.82	2.30	3.11	3.28	<0.001
uPCR (g/gCr)	2.12	7.82	1.20	2.70	5.48	5.69	<0.001
Urinary protein levels <1+	30 (32.6 %)		35 (48.6 %)		18 (13.0 %)		ND**
Urinary OB levels <1+	52 (56.5 %)		54 (75.0 %)		49 (46.2 %)		ND**
Serum Cr (mg/dl)	3.42	2.72	2.34	2.24	1.12	1.13	<0.001
eGFR (ml/min/1.73 m^2^)	24.2	18.7	33.6	20.3	66.6	30.7	<0.001
Serum TP (g/dl)	6.90	1.07	7.10	0.82	5.94	1.12	<0.001
Serum Alb (g/dl)	3.35	0.72	3.96	0.58	2.82	0.95	<0.001
Serum TC (mg/dl)	177.1	46.0	187.5	43.6	274.4	122.0	<0.001
HbA1c (NGSP) (%)	6.14	0.80	5.93	1.27	5.91	0.78	0.017

ATIL, acute tubulointerstitial lesions; CTIL, chronic tubulointerstitial lesions; BP, blood pressure; OB, occult blood level according to the dipstick test; uPCR, urinary protein to creatinine ratio; Cr, creatinine; eGFR, estimated glomerular filtration rate; TP, total protein; Alb, albumin; TC, total cholesterol; HbA1c, glycated hemoglobin

* *p* values were calculated using ANOVA or the Kruskal–Wallis test; ** *ND* not determined

As for hematuria, 195 cases (59.4 %) were found to be (−) or (±) for occult blood in dipstick tests, and 235 cases (70.6 %) were considered to be red blood cell (RBC)-negative or to have <5 RBC/high-powered field (hpf) in their urinary sediments (Table 3). In addition, 52 cases (56.5 %) of ATIL and 54 cases (77.0 %) of CTIL were classified as (−) or (±) for hematuria in dipstick tests (Table 4).

The baseline clinical and laboratory findings of the cases in each of the 3 major pathological categories are shown in Table 4. As mentioned above, the patients with ATIL and CTIL exhibited milder urinary abnormalities than those with glomerular lesions. However, more marked changes in the serum creatinine level and the eGFR were seen in the patients with ATIL or CTIL (ATIL, CTIL, and glomerular lesions: mean serum creatinine levels: 3.42, 2.34, and 1.12 mg/dl, respectively; mean eGFR: 24.2, 33.6, and 66.6 ml/min/1.73 m^2^, respectively; *p* < 0.001). On the other hand, the patients with glomerular lesions exhibited lower serum albumin levels (2.82 ± 0.95 g/dl, *p* < 0.001), higher serum cholesterol levels (274.4 ± 122 mg/dl, *p* < 0.001), and nephrotic range proteinuria (daily proteinuria: 3.11 ± 3.28 g; UPCR: 5.48 ± 5.69 g/gCr, *p* < 0.001).

### The CGA risk classification (heat map) in each pathological category of DIKD

**Fig. 5:**
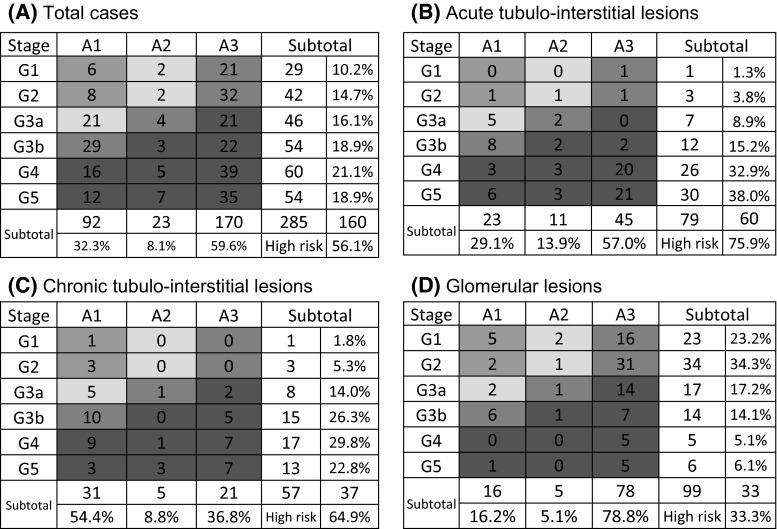
The CGA risk classification of DIKD. According to the CGA risk classification, high-risk (*red* zone) cases accounted for 56.1 % of all cases (**a**), and 75.9 % (**b**), 64.9 % (**c**), and 33.3 % (**d**) of those involving acute tubulointerstitial lesions, chronic tubulointerstitial lesions, and glomerular injuries, respectively (color figure online)

The CGA classification results for each pathological category are shown in Fig. 5 and Supplemental Tables 4 and 5. With regard to the G and A stages of the DIKD patients, stages G4 and A3 were the most common from 2007 and 2015. Furthermore, 123 cases (39.7 %) were considered to involve advanced G stages (G4 or 5), and 175 cases (58.5 %) were classified as stage A3.

The degree of proteinuria detected in the patients’ 24-h urine or spot urine samples increased with the G stage, even among the cases involving AKII and CTIL. According to the CGA risk classification, high-risk (red zone) cases accounted for 56.1 % of all cases of DIKD and 75.9, 64.9, and 33.3 % of cases involving ATIL, CTIL, and glomerular lesions, respectively (Fig. 5a–d).

### The causative drugs in 102 cases

The causative drugs were identified in 102 cases. Of these, bucillamine was the causative drug in 38 cases of MN; calcineurin inhibitors (CNI), such as cyclosporine or tacrolimus, were found to be the causative drug in 27 cases of CTIL or sclerotic lesions; and anticancer drugs were demonstrated to be the causative drugs in 17 cases (including gemcitabine in 3 cases of thrombotic microangiopathy and anti-vascular endothelial growth factor (VEGF) drugs in 3 cases with various pathologies). Anti-neutrophil cytoplasmic antibody (ANCA)-related nephritis, a representative glomerular lesion, was caused by propyl thiouracil (PTU) in 3 cases (Table 5, Supplemental Fig. 3).

**Table 5:** The causative drugs and pathological classifications of 102 cases of drug-induced kidney disease

	Glomerular lesions	ATIL	CTIL	Sclerotic lesions	Others	*n*
Bucillamine	38 (MN)					38
Other DMARD	4					4
CNI	14		2	7	4	27
Anticancer drugs		2	2		7	11
Gemcitabine					3 (TMA)	3
Anti-VEGF drugs	2 (MN, CrGN)				1 (NS)	3^a^
NSAID		4	3			7
PTU	3 (ANCA vasculitis)					3
Antibiotics		2	1			3
Mesalazine		1	1			2
Others					1	1
Subtotal	61	9	9	7	16	102

ATIL, acute tubulointerstitial lesions; CTIL, chronic tubulointerstitial lesions; MN, membranous nephropathy; DMARD, disease modified anti-rheumatic drugs; CNI, calcineurin inhibitor; TMA, thrombotic microangiopathy; VEGF, vascular endothelial growth factor; CrGN, crescentic glomerulonephritis; NS, nephrosclerosis; NSAID, non-steroidal anti-inflammatory drugs; PTU, propyl thiouracil; ANCA, anti-neutrophil cytoplasmic antibody

^a^All cases were registered in the J-RBR after July 2012

## Discussion and comments

### J-RBR/J-KDR and DIKD

In Japan, renal biopsies have been recorded in the J-RBR since 2007, and clinically diagnosed cases of kidney disease in which renal biopsies were not performed have been registered in the J-KDR since 2009 [1, 2]. More than 18 % of cases of DIKD were registered in the J-RBR in 2009 and 2010 [2]; however, only 7 out of 231 cases (3.0 %) of the condition were registered in the J-KDR from 2007 to 2013 (data not shown); thus, the data from the J-RBR are described in this report. It was speculated that the J-RBR contains data about 20–25 % of the renal biopsies performed in Japan. Thus, reports based on the J-RBR are considered to provide representative data of renal biopsied cases for Japan.

### Clinical presentation of DIKD

Among drug-induced kidney injuries, DIKD accounts for 19–26 % of cases of AKI involving hospitalized patients [8]. However, DIKD is often unrecognized because there are no standard clinical definitions for the condition. Our data showed that 12.8 % of DIKD cases were diagnosed based on their pathological findings. Recently, the International Serious Adverse Event Consortium [13] and the working group of Mehta et al. [9] initiated a phenotype standardization project to develop consensus definitions for drug-induced organ toxicities, including DIKD, based on patients’ clinical symptoms. As a result, Mehta et al. proposed 4 phenotypes of DIKD: AKI, glomerular disorders, tubular dysfunction, and nephrolithiasis, and suggested that they are useful for evaluating drug toxicities across various settings [9]. Previous reports (published in 2007–2009) from the study group of the Japanese Ministry of Welfare and Labor showed that among the hospitals at which 47 representative nephrologists worked (the hospitals were located throughout Japan) DIKD accounted for 0.935 % of all admissions [14]. The major causative drugs responsible for the patients’ renal injuries were non-steroidal anti-inflammatory drugs (NSAID) in 25.1 % of cases, anticancer drugs in 18.0 % of cases, antibiotics in 17.5 % of cases, and radiocontrast agents in 5.7 % of cases. In these cases, 54.6 % of the renal injuries were direct renal injuries. Moreover, 36.5 % of the patients did not recover. In the current study, we dealt more severe and complex cases of DIKD, in which the clinicians decided to perform renal biopsies to obtain more accurate pathological diagnoses. Therefore, it is not surprising that there were discrepancies in the patients’ background data between our study population and those of previous studies in which DIKD was diagnosed based on the patients’ clinical symptoms. Thus, we need to obtain up-to-date information about non-renal biopsied DIKD patients based on standardized clinical criteria in future.

### Pathology of DIKD and newer drugs

Previous studies in which DIKD was diagnosed based on the patients’ clinical symptoms obtained quite different findings regarding the frequencies of each clinical category and causative drug among DIKD patients than the present study. However, studies in which DIKD was diagnosed based on the patients’ pathological findings reported similar pathological data regarding the patients’ categories. Since the 1980s, it has been recognized that drug-induced acute interstitial nephritis associated with methicillin or other penicillins, diuretics, or NSAID commonly presents as acute renal failure [15]. In addition, tubulointerstitial lesions such as acute tubular necrosis can be caused by directly tubulo-toxic drugs (e.g., cisplatin, gentamicin, etc.), which induce minimal glomerular histological changes in the human kidney. In toxicological screening programs for nephrotoxic substances, it was suggested that some types of drug-induced renal injuries are mediated by immune mechanisms; i.e., immune complex glomerular disease and nil disease (a minor glomerular abnormality) [16]. In the past decade, we have identified other glomerular lesions such as collapsing focal segmental glomerulosclerosis, which can be caused by pamidronate [17] and other drugs. Finally, a number of newer therapies such as anti-VEGF therapy have emerged as causative agents of renal toxicity, which produce a variety of pathological changes in the kidney [18, 19]. Some drugs can cause irreversible changes and even end-stage renal disease [20, 21]. The present study included some cases of DIKD involving these new drugs, such as gemcitabine, PTU, and anti-VEGF drugs, and their associated pathological findings. In addition, it was demonstrated that bucillamine is a major cause of DIKD-associated glomerular lesions in Japan. Cases of MN involving a different IgG subclass from idiopathic MN have been described in the literature, and such renal changes are considered to be characteristic bucillamine-induced lesions [22, 23] (Supplemental Table 6). However, the precise mechanisms responsible for bucillamine-induced MN remain unclear.

### Aging and DIKD

Our analysis of the J-RBR revealed that in Japan DIKD mainly affects the elderly. There are several possible reasons for this: (1) the elderly are exposed to drugs more frequently than younger individuals, (2) the elderly are administered inappropriate doses of nephrotoxic drugs that have not been adjusted for age-related renal and systemic changes, (3) the use of serum Cr levels for diagnostic purposes might be of limited use in the elderly, and (4) there is a lower chance of renal recovery in older patients. It is well known that the risk of developing AKI is significantly increased in the elderly for various reasons, e.g., they are at greater risk of drug toxicities, such as contrast medium-induced nephropathy [24].

As concern to ATIL in the elderly, Muriithi et al. reported recently that drug-induced acute interstitial nephritis (AIN) (87 vs. 64 %), especially proton pump inhibitor (PPI)-induced AIN (18 vs. 6 %), was observed significantly more in the elderly compared with younger patients. Moreover, the elderly had higher prevalence of baseline CKD, higher peak creatinine, and more need for dialysis. Thus, the vast majority of AIN cases in the elderly are due to drugs, primarily owing to PPI and antibiotics in the United State [25].

Moreover, the frequencies of nephrotic syndrome-related glomerular diseases caused by immune complex diseases, such as idiopathic MN and rapidly progressive glomerulonephritis caused by ANCA-positive vasculitis are increasing in Japan [4]. Aging might affect the immune responses of the elderly. Thus, the elderly might be more sensitive to drugs, e.g., they might be more susceptible to drug-induced nephrotoxicity or exhibit hypersensitive immune responses to drugs.

### Limitations of this study

1. We cannot exclude the possibility that the J-RBR is subject to sampling bias; however, the J-RBR represents the largest renal biopsy series of elderly (aged over 65 years) and very elderly (over 75 years old) patients in the world [4]. Thus, it is likely to be reasonably representative of the nationwide situation of renal biopsied cases in Japan.
2. We could not examine the outcomes of the DIKD patients in this study. Further studies of the clinical outcomes of DIKD will be necessary.
3. There are no standard definitions for drug-induced nephrotoxicity based on patients’ clinical symptoms. We need to develop clinical strategies for diagnosing and managing DIKD.

## Conclusion

Our analysis of the J-RBR revealed that DIKD mainly affects elderly people in Japan. In addition, approximately half of the renal biopsied cases involved ATIL or CTIL, and roughly one-third involved glomerular lesions, mainly MN and clinical nephrotic syndrome. In future studies, we need to examine the outcomes of DIKD and develop clinical strategies for managing the condition.

## Electronic supplementary material

Below is the link to the electronic supplementary material.

Supplementary material 1 (DOCX 44 kb)

Supplementary material 2 (DOCX 37 kb)

Supplementary material 3 (PPTX 85 kb)
