# Supplementary material for: Drug-induced kidney disease: a study of the Japan Renal Biopsy Registry from 2007 to 2015
Source: Clin Exp Nephrol. 2015 Nov 21;20(5):720–30. doi: 10.1007/s10157-015-1201-4 (PMC5050234; doi:10.1007/s10157-015-1201-4)
Supplement: Supplementary file 1 — Supplementary material 1 (DOCX 44 kb) [file 10157_2015_1201_MOESM1_ESM.docx]

**Supplemental Figures Legends and Tables**

**Supplemental Figure Legends**

Supplemental Figure 1: Pathological diagnoses of the examined cases of drug-induced kidney disease

Supplemental Figure 2: The frequency of drug-induced kidney disease in each pathological category according to age

Supplemental Figure 3: The causative drugs of drug-induced kidney disease in 102 cases

**Supplemental Tables**

Supplemental Table 1: The pathological categories of the examined cases of drug-induced kidney disease

|  | First period* | % | Second period** | % | Total | % |
| --- | --- | --- | --- | --- | --- | --- |
| Acute tubulointerstitial lesions | 59 | 26.8 | 28 | 25.9 | 87 | 26.5 |
| Chronic tubulointerstitial lesions | 55 | 25.0 | 17 | 15.7 | 72 | 22.0 |
| Glomerular lesions | 65 | 29.5 | 40 | 37.0 | 105 | 32.0 |
| Sclerotic lesions | 17 | 7.7 | 1 | 0.9 | 18 | 5.5 |
| Others + transplanted kidney | 24 | 10.9 | 22 | 20.4 | 46 | 14.0 |
| Total | 220 | 100 | 108 | 100 | 328 | 100 |

See Table 2; *: July 2007- June 2012; **: July 2012-June 2015

Supplemental Table 2: Numbers of registered cases of drug-induced kidney disease according to age

| Age | First period* | % | Second period** | % | Total | % |
| --- | --- | --- | --- | --- | --- | --- |
| <10 | 3 | 1.4 | 2 | 1.9 | 5 | 1.5% |
| 10 - 19 | 11 | 5.0 | 6 | 5.6 | 17 | 5.2% |
| 20 - 29 | 16 | 7.3 | 8 | 7.5 | 24 | 7.3% |
| 30 - 39 | 19 | 8.6 | 4 | 3.7 | 23 | 7.0% |
| 40 - 49 | 30 | 13.6 | 14 | 13.1 | 44 | 13.5% |
| 50 - 59 | 38 | 17.3 | 16 | 15.0 | 54 | 16.5% |
| 60 - 69 | 55 | 25.0 | 34 | 31.8 | 89 | 27.2% |
| 70 - 79 | 40 | 18.2 | 18 | 16.8 | 58 | 17.7% |
| 80+ | 8 | 3.6 | 5 | 4.7 | 13 | 4.0% |
| Total | 220 | 100.0 | 107*** | 100.0 | 327*** | 100.0% |

See Figure 2; *: July 2007- June 2012; **: July 2012-June 2015; ***: the data for one case was lost

Supplemental Table 3: The frequency of drug-induced kidney disease in the J-RBR

| Age | Cases | % |
| --- | --- | --- |
| <10 | 5 | 0.53 |
| 10 - 19 | 17 | 0.62 |
| 20 - 29 | 24 | 0.82 |
| 30 - 39 | 23 | 0.64 |
| 40 - 49 | 44 | 1.26 |
| 50 - 59 | 54 | 1.40 |
| 60 - 69 | 89 | 1.86 |
| 70 - 79 | 58 | 1.70 |
| 80 - 89 | 13 | 1.78 |
| Total | 327* | 1.24 |

See Figure 3; *: the data for one case was lost

Supplemental Table 4: CGA classification G stage of the cases of drug-induced kidney disease in each pathological category

| G stage  Pathological category | G1 | G2 | G3a | G3b | G4 | G5 | Sub  total | % |
| --- | --- | --- | --- | --- | --- | --- | --- | --- |
| Acute tubulointerstitial lesions | 1 | 3 | 7 | 13 | 30 | 33 | 87 | 28.1 |
| Chronic tubulointerstitial lesions | 1 | 5 | 11 | 21 | 19 | 13 | 70 | 22.6 |
| Glomerular lesions | 23 | 34 | 17 | 14 | 5 | 6 | 99 | 31.9 |
| Sclerotic lesions | 2 | 2 | 4 | 4 | 4 | 2 | 18 | 5.8 |
| Others | 3 | 0 | 11 | 11 | 8 | 3 | 36 | 11.6 |
| Subtotal | 30 | 44 | 50 | 63 | 66 | 57 | 310 | 100 |
| % | 9.7 | 14.2 | 16.1 | 20.3 | 21.3 | 18.4 | 100 |  |

Supplemental Table 5: CGA classification A stage of the cases of drug-induced kidney disease in each pathological category

| A stage  Pathological category | A1 | A2 | A3 | Subtotal | % |
| --- | --- | --- | --- | --- | --- |
| Acute tubulointerstitial lesions | 23 | 14 | 46 | 83 | 27.8 |
| Chronic tubulointerstitial lesions | 33 | 5 | 21 | 59 | 19.7 |
| Glomerular lesions | 18 | 6 | 80 | 104 | 34.8 |
| Sclerotic lesions | 7 | 1 | 8 | 16 | 5.4 |
| Others | 16 | 1 | 20 | 37 | 12.4 |
| Subtotal | 97 | 27 | 175 | 299 | 100 |
| % | 32.4 | 9.0 | 58.5 | 100 |  |

Supplemental Table 6: Reports about bucillamine-related glomerular disease in Japan

| Year | First Author | Journal |
| --- | --- | --- |
| 2015 | Manabe S, et al. | Case Rep Nephrol Dial. 2015; 5(1):30-8. |
| 2006 | Hoshino J, et al. | Nephron Clin Pract. 2006; 104(1):c15-9. |
| 2004 | Ohno I | Nihon Rinsho. 2004; 62(10):1919-24. Review. Japanese. |
| 2004 | Ohtani H, et al. | Nephrol Dial Transplant. 2004; 19(3):574-9. |
| 2003 | Obayashi M, et al. | Clin Exp Nephrol. 2003; 7(4):275-8. |
| 2002 | Nagahama K, et al. | Am J Kidney Dis. 2002; 39(4):706-12. |
| 2000 | Kawasaki Y, et al. | Pediatr Int. 2000; 42(3):316-8. |
| 1999 | Oshitani N, et al. | Clin Exp Pharmacol Physiol. 1999; 26(12):956-8. |
| 1998 | Nakanishi K, et al. | Nihon Jinzo Gakkai Shi. 1998; 40(8):607-11. Japanese. |
| 1994 | Yoshida A, et al. | Clin Ther. 1994; 16(6):1000-6. |
| 1993 | Kikuchi M, et al. | Ryumachi. 1993; 33(3):215-22. Japanese. |
| 1992 | Shimokama T, et al. | Nihon Jinzo Gakkai Shi. 1992;34(3):301-7. Japanese. |
| 1991 | Baba N, et al. | Nihon Jinzo Gakkai Shi. 1991; 33(6):629-34. Japanese. |
| 1991 | Yoshida A, et al. | Am J Nephrol. 1991; 11(4):284-8. |
| 1990 | Ogawa N, et al. | Ryumachi. 1990; 30(5):362-6, 308. Japanese. |
| 1990 | Kawano M, et al. | Nihon Jinzo Gakkai Shi. 1990;32(7):817-21. Japanese. |
