# Supplementary material for: Drug-induced kidney disease: a study of the Japan Renal Biopsy Registry from 2007 to 2015
Source: Clin Exp Nephrol. 2015 Nov 21;20(5):720–30. doi: 10.1007/s10157-015-1201-4 (PMC5050234; doi:10.1007/s10157-015-1201-4)
Supplement: Supplementary file 2 — Supplementary material 2 (DOCX 37 kb) [file 10157_2015_1201_MOESM2_ESM.docx]

**Appendix**

The following investigators and initial institutions have participated in the development of the J-RBR since 2007: Hirofumi Makino and Hitoshi Sugiyama (Okayama University), *late* Takashi Taguchi (Nagasaki University), Hitoshi Yokoyama (Kanazawa Medical University), Hiroshi Sato (Tohoku University), Takao Saito and Yoshie Sasatomi (Fukuoka University), Yukimasa Kohda (Kumamoto University; present institution: Hikarinomori Clinic), Shinichi Nishi (Niigata University; present institution: Kobe University), Kazuhiko Tsuruya and Yutaka Kiyohara (Kyushu University), Hideyasu Kiyomoto (Kagawa University; present institution: Tohoku Medical Megabank Organization, Tohoku University), Hiroyuki Iida (Toyama Prefectural Central Hospital; present institution: Saiseikai Takaoka Hospital ), Tamaki Sasaki (Kawasaki Medical School), Makoto Higuchi (Shinshu University; present institution: National Hospital Organization Matsumoto Medical Center (Matsumoto) ), Motoshi Hattori (Tokyo Women’s Medical University), Kazumasa Oka (Osaka Kaisei Hospital; present institution: Hyogo Prefectural Nishinomiya Hospital), Shoji Kagami (The University of Tokushima Graduate School), Michio Nagata (University of Tsukuba), Tetsuya Kawamura (The Jikei University School of Medicine), Masataka Honda (Tokyo Metropolitan Children’s Medical Center), Yuichiro Fukasawa (KKR Sapporo Medical Center ; present institution: Sapporo City General Hospital), Atsushi Fukatsu (Kyoto University Graduate School of Medicine; present institution: Yachiyo Hospital), Kunio Morozumi (Japanese Red Cross Nagoya Daini Hospital; present institution: Masuko Memorial Hospital ), Norishige Yoshikawa (Wakayama Medical University), Yukio Yuzawa (Fujita Health University), Seiichi Matsuo (Nagoya University Graduate School of Medicine) and Kensuke Joh (Chiba-East National Hospital; present institution: Tohoku University Graduate School of Medicine).

**Hokkaido District**

● Asahikawa Medical University Hospital (Division of Cardiology, Nephrology, Pulmonology and Neurology, Department of Internal Medicine), Naoyuki Hasebe, Naoki Nakagawa, Junko Chinda

● National Hospital Organization Hokkaido Medical Center (Department of Nephrology), Sekiya Shibazaki, Tomotsune Miyamoto, Masanori Ito

● Hokkaido University Graduate School of Medicine (Department of Medicine II), Saori Nishio

● Hokkaido University Graduate School of Medicine (Department of Pediatrics),

Takayuki Okamoto, Yasuyuki Sato, Takeshi Yamazaki

● KKR Sapporo Medical Center (Department of Pathology), Akira Suzuki

**Tohoku District**

●Iwate Prefectural Central Hospital (Department of Nephrology), Jun Soma, Izaya Nakaya, Noriyo Sasaki

● Fukushima Medical University School of Medicine (Department of Nephrology, Hypertension, Diabetology, Endocrinology, and Metabolism), Masaaki Nakayama, Koichi Asahi, Hiroaki Satoh

●Japan Community Health care Organization Sendai Hospital (Department of Nephrology), Toshinobu Sato, Asako Fujimori, Satoru Sanada, Mitsuhiro Sato

● Tohoku University Hospital and affiliated hospitals (Internal Medicine), Hiroshi Sato, Mariko Miyazaki, Takashi Nakamichi, Tae Yamamoto

● Yamagata University School of Medicine (Department of Cardiology, Pulmonology, and Nephrology), Tsuneo Konta, Kazunobu Ichikawa, Kazuko Suzuki

● Yamagata University School of Medicine (Department of Pediatrics), Daisuke Ogino

**Kanto District**

●National Hospital Organization Chiba-East Hospital (Department of Pathology), Hiroshi Kitamura, (Department of Internal Medicine),Toshiyuki Imazawa, (Department of Pediatrics), Chieko Matsumura,(Department of Surgery), Naotake Akutsu, (Department of Urology), Koichi Kamura

● Dokkyo Medical University Koshigaya Hospital (Department of Nephrology), Tetsuro Takeda

● Dokkyo Medical University (Department of Cardiology and Nephrology), Toshihiko Ishimitsu

● Gunma University Graduate School of Medicine (Department of Science and Clinical Medicine), Yoshihisa Nojima, Keiju Hiromura

● Jichi Medical University (Division of Nephrology), Daisuke Nagata, Shigeaki Muto, Osamu Saito, Tetsu Akimoto

● The Jikei University School of Medicine (Division of Nephrology and Hypertension)

Takashi Yokoo, Nobuo Tsuboi

● The Jikei University School of Medicine, Katsushika Medical Center (Division of Nephrology and Hypertension), Masato Ikeda, Shinya Yokote

● The Jikei University School of Medicine, Daisan Hospital (Division of Nephrology and Hypertension), Kazushige Hanaoka, Kentaro Koike, Haruko Suetsugu, Mai Tanaka

●The Jikei University Kashiwa Hospital (Division of Nephrology and Hypertension), Makoto Ogura, Akihiko Hamaguchi, Yukio Maruyama, Hideyuki Itoh

●Juntendo University Faculty of Medicine (Division of Nephrology, Department of Internal Medicine), Yusuke Suzuki, Miyuki Takagi, Chieko Hamada, Satoshi Horikoshi

● Kawaguchi Municipal Medical Center (Division of Nephrology), Masahiro Ishikawa

● Kyorin University School of Medicine (Department of Urology), Kikuo Nutahara

● Kyorin University School of Medicine (Division of Nephrology and Rheumatology, First Department of Internal Medicine), Yoshihiro Arimura, Shinya Kaname

● Mito Saiseikai General Hospital (Division of Nephrology), Itaru Ebihara, Chihiro Satho

● Nippon Medical School (Division of Nephrology, Department of Internal Medicine), Shuichi Tsuruoka, Yukinao Sakai, Akio Hirama

● Nihon University School of Medicine (Division of Nephrology, Hypertension and Endocrinology), Yoshinobu Fuke

● Saitama Medical University, Faculty of Medicine (Department of Nephrology), Hirokazu Okada, Hiromichi Suzuki, Tsutomu Inoue

● Saitama Medical University, Saitama Medical Center (Department of Nephrology and Hypertension), Takatsugu Iwashita, Yuuta Kogure, Kouichi Kanouzawa, Hajime Hasegawa

● Showa University School of Medicine (Division of Nephrology), Aki Kuroki

● Showa University Fujigaoka Hospital (Division of Nephrology), Yoshihiko Inoue

● St. Marianna University School of Medicine (Division of Nephrology and Hypertension, Department of Internal Medicine), Daisuke Ichikawa, Sayuri Shirai, Yugo Shibagaki

● Tokai University School of Medicine (Division of Nephrology, Endocrinology and Metabolism), Masayuki Endoh, Hisae Tanaka

● Teikyo University School of Medicine (Department of Internal Medicine), Shunya Uchida

● Teikyo University School of Medicine (Department of Urology), Shigeo Horie, Satoru Muto

● Tokyo Medical University Ibaraki Medical Center (Department of Nephrology), Masaki Kobayashi, Kouichi Hirayama, Homare Shimohata

● Tokyo Metropolitan Children's Medical Center (Department of Nephrology), Hiroshi Hataya

● Tokyo Women's Medical University (Department of Pediatric Nephrology), Motoshi Hattori, Kiyonobu Ishizuka, Noriko Sugawara

● Tokyo Women's Medical University (The Forth Department of Medicine), Kosaku Nitta, Keiko Uchida, Takahito Moriyama

● Toranomon Hospital, Nephrology Center, Yoshifumi Ubara, Tatsuya Suwabe, Junichi Hoshino, Noriko Hayami

● University of Tokyo Hospital (Department of Hemodialysis & Apheresis), Norio Hanafusa

● University of Tokyo（Department of Nephrology and Endocrinology）, Toshiro Fujita, Masaomi Nangaku, Takehiko Wada

● University of Tokyo（Department of Pediatrics), Yutaka Harita, Kenichiro Miura

● University of Tsukuba, Faculty of Medicine, (Department of Nephrology), Kunihiro Yamagata, Joichi Usui, Tetsuya Kawamura

● Yokohama City University Graduate School of Medicine and School of Medicine (Department of Medical Science and Cardiorenal Medicine), Satoshi Umemura, Masato Oosawa

● Yokohama City University Medical Center, Nobuhito Hirawa, Keisuke Yatsu, Yuichiro Yamamoto, Sanae Saka

**Koushinetsu District**

● Niigata University Graduate School of Medical and Dental Sciences (Division of Clinical Nephrology and Rheumatology), Ichiei Narita, Shin Goto, Yumi Itoh, Naofumi Imai

● Shinshu University School of Medicine (Division of Nephrology), Yuji Kamijo, Koji Hashimoto, Makoto Harada, Akinori Yamaguchi, Yosuke Yamada

●University of Yamanashi Hospital (Third Department of Internal Medicine), Fumihiko Furuya, Daiichiro Akiyama, Kazuya Takahashi, Ayako Okamura

**Hokuriku District**

● National Hospital Organization Kanazawa Medical Center (Department of Nephrology and　Rheumatology), Kiyoki Kitagawa

● Kanazawa Medical University School of Medicine (Department of Nephrology),　Hitoshi Yokoyama, Keiji Fujimoto, Junko Imura, Norifumi Hayashi

● Kanazawa Medical University (Department of Diabetology & Endocrinology), Daisuke Koya, Yuka Kurosima,

● Kanazawa University Hospital (Division of Nephrology), Takashi Wada, Kengo Furuichi, Miho Shimizu, Tadashi Toyama

● Komatsu Sophia Hospital, Yasuhiro Katou, Taito Miyake

● Moriyama Koshino Clinic, Yoshitaka Koshino

● Pubulic Central Hospital of Matto-Ishikawa, Kazuya Takasawa, Chikako Takaeda

● Sugita Genpaku Memorial Obama Municipal Hospital, Haruyoshi Yoshida, Takayasu Horiguchi

● Toyama Prefectural Central Hospital (Department of Internal Medicine), JunyaYamahana, Masahiko Kawabata

● Toyama City Hospital(Department of Internal Medicine), Satoshi Ota, Yoh-ichi Ishida

● University of Fukui, Faculty of Medical Sciences (Division of Nephrology, Department of General Medicine), Masayuki Iwano, Hideki Kimura, Naoki Takahashi, Kenji Kasuno

● University of Toyama (Second Department of Internal Medicine), Fumihiro Tomoda

**Tokai District**

● Aichi Children's Health and Medical Center (Department of Pediatric Nephrology), Osamu Uemura, Satoshi Yamakawa

● Aichi Medical University School of Medicine (Division of Nephrology and Rheumatology), Keisuke Suzuki, Takuhito Nagai, Naoto Miura, Hirokazu Imai

● Chuno Kosei Hospital, Shogo Kimura, Yuka Soga

● Fujinomiya City General Hospital, Masanori Sakakima, Kazuto Kitajima, Taichi Sato, Yutaro Kawakatsu

● Fujita Health University School of Medicine (Department of Nephrology), Yukio Yuzawa, Satoshi Sugiyama

● Hamamatsu University School of Medicine, University Hospital (Internal Medicine1, Division of Nephrology), Hideo Yasuda, Naro Ohashi, Takamasa Iwakura

● Japanese Red Cross Nagoya Daini Hospital (Kidney Center), Asami Takeda, Yasuhiro Otsuka

● Nagoya City East Medical Center, Minamo Ono, Tatsuya Tomonari

● Nagoya City University Graduate School of Medical Sciences (Department of Cardio-Renal Medicine and Hypertension), Michio Fukuda, Toshiyuki Miura, Yoshiaki Ogiyama, Ryo Sato

● Nagoya Kyoritsu Hospital (Department of Internal Medicine), Hirotake Kasuga

● Nagoya University Graduate School of Medicine (Department of Nephrology), Seiichi Matsuo, Shoichi Maruyama, Yoshinari Yasuda

● Shizuoka General Hospital (Department of Nephrology), Noriko Mori, Satoshi Tanaka

● Mie University Graduate School of Medicine (Department of Cardiology and Nephrology), Eiji Ishikawa, Mika Fujimoto, Tomohiro Murata, Masaaki Ito

● Yokkaichi Social Insurance Hospital (Division of Nephrology and Blood Purification), Yasuhide Mizutani, Hitoshi Kodera, Masato Miyake

**Kinki District**

● Hyogo Prefectural Nisihinomiya Hospital (Department of Pathology), Kazumasa Oka

● Ikeda City Hospital (Division of Nephrology), Nobuyuki Kajiwara

● Kitano Hospital, The Tazukekofukai Medical Research Institute (Division of Nephrology and Dialysis), Eri Muso, Kazuo Tosikoshi, Tomomi Endo, Yukako Iwasaki

● Kobe University Graduate School of Medicine (Division of Nephrology and Kidney Center), Shinichi Nishi, Shunske Goto

● Kobe University Graduate School of Medicine (Department of Pediatrics), Kazumoto Iijima, Hiroshi Kaito, Takeshi Ninchoji

● JCHO Kobe Central Hospital, Yoko Adachi

● National Hospital Organization Kyoto Medical Center (Division of Nephrology), Koichi Seta, Kensei Yahata

● Kyoto Prefectural University of Medicine Graduate School of Medical Science (Department of Nephrology), Keiichi Tamagaki, Tetsuro Kusaba

● Kyoto University Graduate School of Medicine (Department of Nephrology), Motoko Yanagita, Tatsuo Tsukamoto, Takeshi Matsubara, Hideki Yokoi, Kaoru Sakai, Akira Ishii

● Nara Medical University (First Department of Internal Medicine), Yoshihiko Saito, Kenichi Samejima

● National Cerebral and Cardiovascular Center (Division of Hypertension and Nephrology), Satoko Nakamura

● Osaka City University Graduate School of Medicine (Department of Nephrology), Eiji Ishimura, Ikue Kobayashi, Mitsuru Ichii, Yoshiteru Ohno

● Osaka City General Hospital (Division of Nephrology and Hypertension), Masahito Imanishi, Takashi Morikawa, Chizuko Kitabayashi, Yoshio Konishi

● Osaka General Medical Center (Department of Kidney Disease and Hypertension), Terumasa Hayashi, Tatsuya Shoji

●Osaka Medical Center and Research Institute for Maternal and Child Health (Department of Pediatric Nephrology and Metabolism), Kenichi Satomura

● Osaka Red Cross Hospital (Department of Nephrology), Akira Sugawara, Masao Koshikawa, Yoshihisa Ogawa, Tomoko Kawanishi

● Osaka University Graduate School of Medicine (Department of Geriatric Medicine and Nephrology), Yoshitaka Isaka, Ryohei Yamamoto

● Saiseikai Shiga Hospital (Division of Nephrology), Toshiki Nishio

● Shiga University of Medical Science (Department of Medicine), Shinichi Araki

● Shirasagi Hospital (Kidney Center), Shigeichi Shoji, Kenjiro Yamakawa, Senji Okuno

● Toyonaka Municipal Hospital (Division of Nephrology), Megumu Fukunaga

● Wakayama Medical University (Department of Pediatrics), Norishige Yoshikawa, Koichi Nakanishi, Yuko Shima

● Wakayama Medical University (Division of Nephrology, Department of Internal Medicine), Takashi Shigematsu, Masaki Ohya

**Chugoku District**

● Kawasaki Medical School (Department of Nephrology and Hypertension), Naoki Kashihara,Tamaki Sasaki, Sohachi Fujimoto

● Kurashiki Central Hospital (Division of Nephrology), Kenichiro Asano, Masaru　Kinomura

● Hiroshima University Hospital (Department of Nephrology), Takao Masaki, Shigehiro Doi, Ayumu Nakashima, Toshinori Ueno

● Mizushima Kyodo Hospital (Department of Internal Medicine), Nobuyoshi Sugiyama, Yuichirou Inaba, Kan Yamazaki, Kouji Ozeki

● Okayama Saiseikai General Hospital (Department of Nephrology), Makoto Hiramatsu, Keisuke Maruyama, Noriya Momoki

● Okayama University Graduate School of Medicine, Dentistry, and Pharmaceutical Sciences (Department of Medicine and Clinical Science),Tatsuyuki Inoue, Keiko Tanaka, Ayu Akiyama

● Saiseikai Yamaguchi General Hospital (Department of Internal Medicine), Tsuyoshi Imai

● Shimane University Faculty of Medicine (Division of Nephrology), Takafumi Ito

● Tottori University, Faculty of Medicine (Division of Pediatrics and Perinatology), Shinichi Okada, Yasuo Kawaba, Koichi Kitamoto, Hiroki Yokoyama

**Shikoku District**

● Kagawa University, Faculty of Medicine (Department of Cardiorenal and Cerebrovascular Medicine & Department of Clinical Pathology), Masakazu Kohno, Yoko Nishijima, Tadashi Hara,Yoshio Kushida

● Kochi University, Kochi Medical School (Department of Endocrinology, Metabolism and Nephrology), Yoshio Terada, Toru Kagawa, Taro Horino, Yoshiko Shimamura

● Kochi University, Kochi Medical School (Department of Pediatrics), Mikiya Fujieda, Masayuki Ishihara

● Tokushima University Graduate School (Department of Pediatrics, Institute of Biomedical Sciences), Shoji Kagami, Maki Urushihara, Yukiko Kinoshita

● Tokushima University Graduate School (Department of Nephrology), Toshio Doi, Hideharu Abe, Kojiro Nagai

**Kyushu District**

● Fukuoka University School of Medicine (Division of Nephrology & Rheumatology, Departments of Internal Medicine), Takao Saito, Yoshie Sasatomi, Satoru Ogahara, Satoshi Hisano

● Japanese Red Cross Fukuoka Hospital (Department of Pediatrics), Ken Hatae, Maiko Hinokiyama, Rie Kuroki, Hiroyo Maruyama

● Japanese Red Cross Fukuoka Hospital (Nephrology and Dialysis Center), Hideki Hirakata, Koji Mitsuiki

● Kumamoto University Graduate School of Medical Sciences (Department of Nephrology), Masashi Mukoyama, Masataka Adachi, Takashige Kuwabara

● Jinseikai Clinic Hikarinomori, Yukimasa Kohda

● Kurume University School of Medicine (Division of Nephrology, Department Medicine), Seiya Okuda, Daisuke Wakasugi, Kiyomi Koike

● Kyushu University Graduate School of Medical Sciences (Department of Medicine and Clinical Science ),　Kazuhiko Tsuruya, Shunsuke Yamada, Akihiro Tsuchimoto, Hisako Yoshida

● Kyushu University Graduate School of Medical Sciences (Department of Environmental Medicine), Yutaka Kiyohara, Toshiharu Ninomiya, Masaharu Nagata

● Miyazaki Prefectural Miyazaki Hospital (Division of Nephrology), Naoko Yokota-Ikeda, Shigehiro Uezono, Keiko Kodama

● Nagasaki University Hospital (Department of Pathology), *late* Takashi Taguchi

● Nagasaki University Hospital (Second Department of Internal Medicine), Tomoya　Nishino, Hideyuki Arai, Yoko Obata, Tadashi Uramatsu

● National Fukuoka Higashi Medical Center (Kidney Unit), Ritsuko Katafuchi

● National Kyushu Medical Center Hospital, Masaru Nakayama

● Oitaken Kouseiren Tsurumi Hospital (Division of Nephrology), Ryokichi Yasumori

● Saga University, Faculty of Medicine (Department of Internal Medicine), Yuji Ikeda, Motoaki Miyazono, Tomoya Kishi, Tsuyoshi Takashima, Syuichi Rikitake

● St. Mary's Hospital, Harumichi Higashi

● University of Miyazaki Hospital (First Department of Internal Medicine), Shouichi Fujimoto, Yuji Sato, Masao Kikuchi

● University of Occupational and Environmental Health (Second Department of Internal Medicine), Masahito Tamura,Tetsu Miyamoto

● University of the Ryukyus Graduate School of Medicine (Department of Cardiology, Nephrology and Neurology), Yusuke Ohya, Kentaro Kohagura
