# Supplementary material for: Drug-induced kidney disease: a study of the Japan Renal Biopsy Registry from 2007 to 2015
Source: Clin Exp Nephrol. 2015 Nov 21;20(5):720–30. doi: 10.1007/s10157-015-1201-4 (PMC5050234; doi:10.1007/s10157-015-1201-4)
Supplement: Supplementary file 3 — Supplementary material 3 (PPTX 85 kb) [file 10157_2015_1201_MOESM3_ESM.pptx]

## Slide 1
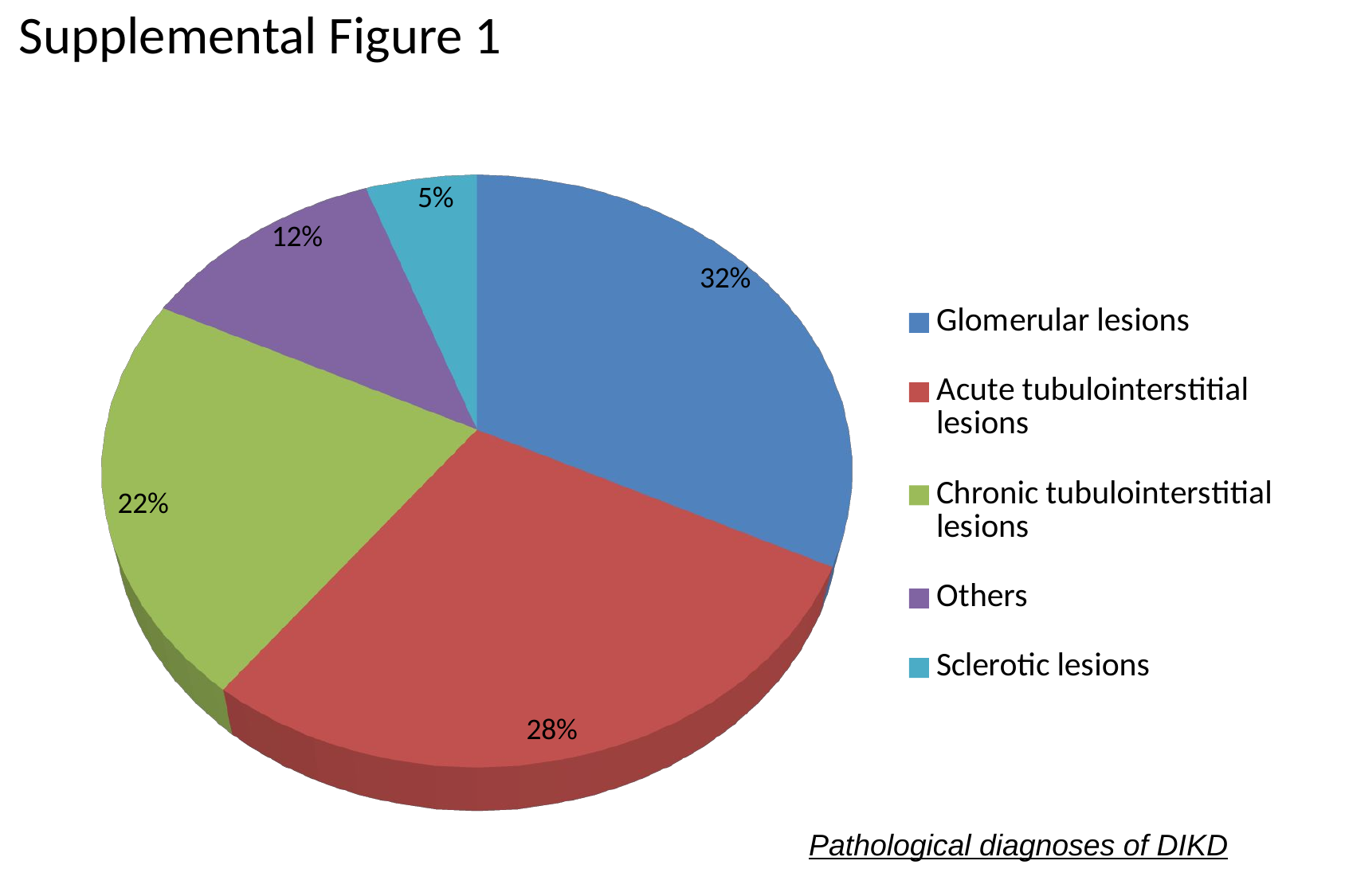

Supplemental Figure 1
[unsupported chart]
Pathological diagnoses of DIKD

## Slide 2
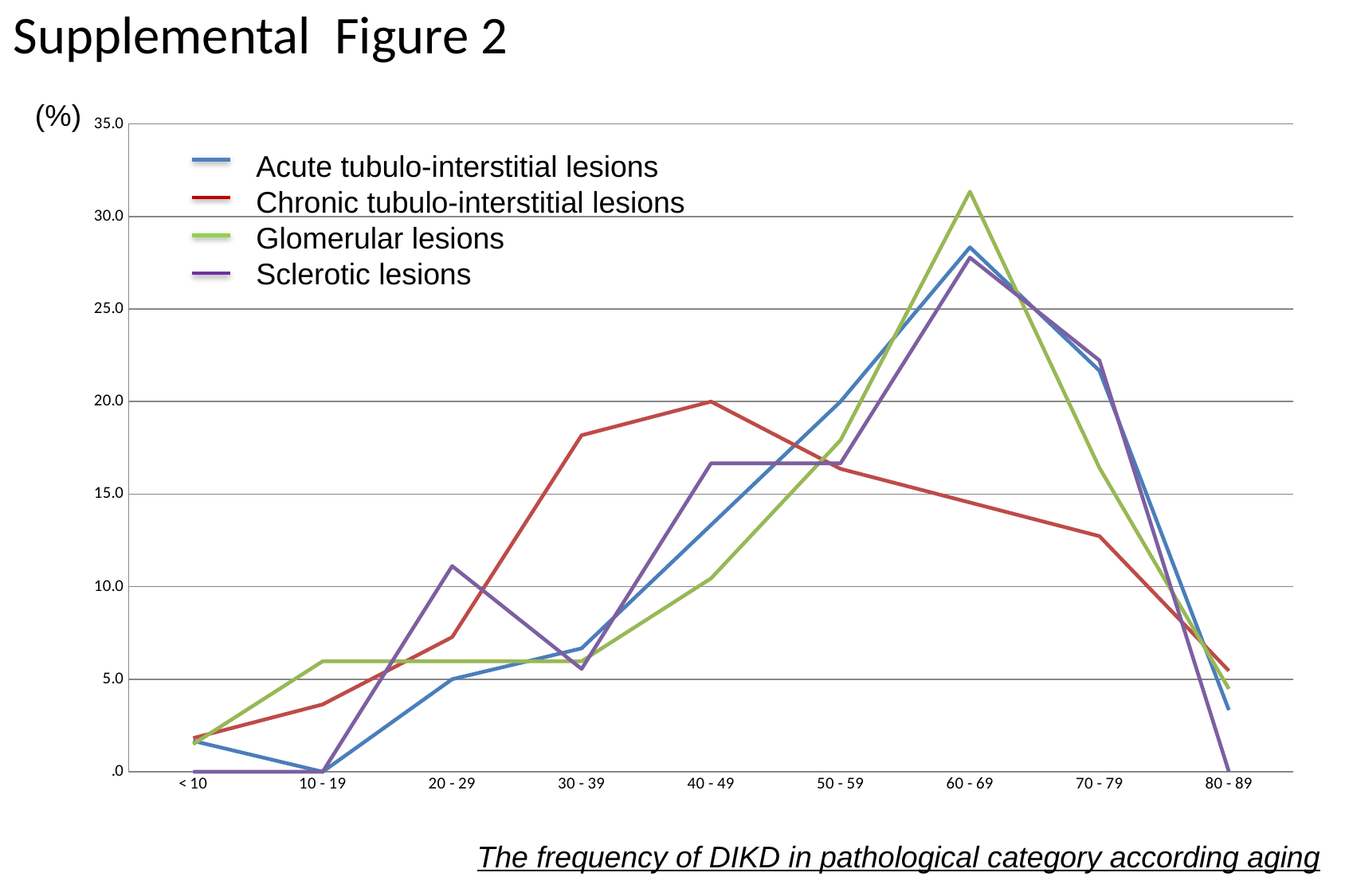

Supplemental Figure 2
(%)
### Chart
| Category | Ⅰ．急性間質性腎疾患 | Ⅱ．慢性間質性腎疾患 | Ⅲ．糸球体疾患 | Ⅳ．硬化性変化 |
|---|---|---|---|---|
| < 10 | 1.6666666666666667 | 1.8181818181818181 | 1.492537313432836 | 0.0 |
| 10 - 19 | 0.0 | 3.6363636363636354 | 5.970149253731346 | 0.0 |
| 20 - 29 | 5.0 | 7.2727272727272725 | 5.970149253731346 | 11.111111111111105 |
| 30 - 39 | 6.666666666666667 | 18.181818181818194 | 5.970149253731346 | 5.555555555555552 |
| 40 - 49 | 13.333333333333334 | 20.0 | 10.447761194029848 | 16.666666666666668 |
| 50 - 59 | 20.0 | 16.363636363636356 | 17.91044776119403 | 16.666666666666668 |
| 60 - 69 | 28.333333333333318 | 14.545454545454549 | 31.343283582089544 | 27.77777777777778 |
| 70 - 79 | 21.666666666666668 | 12.72727272727272 | 16.417910447761187 | 22.222222222222207 |
| 80 - 89 | 3.3333333333333335 | 5.4545454545454515 | 4.477611940298507 | 0.0 |Acute tubulo-interstitial lesions
Chronic tubulo-interstitial lesions
Glomerular lesions
Sclerotic lesions
The frequency of DIKD in pathological category according aging

## Slide 3
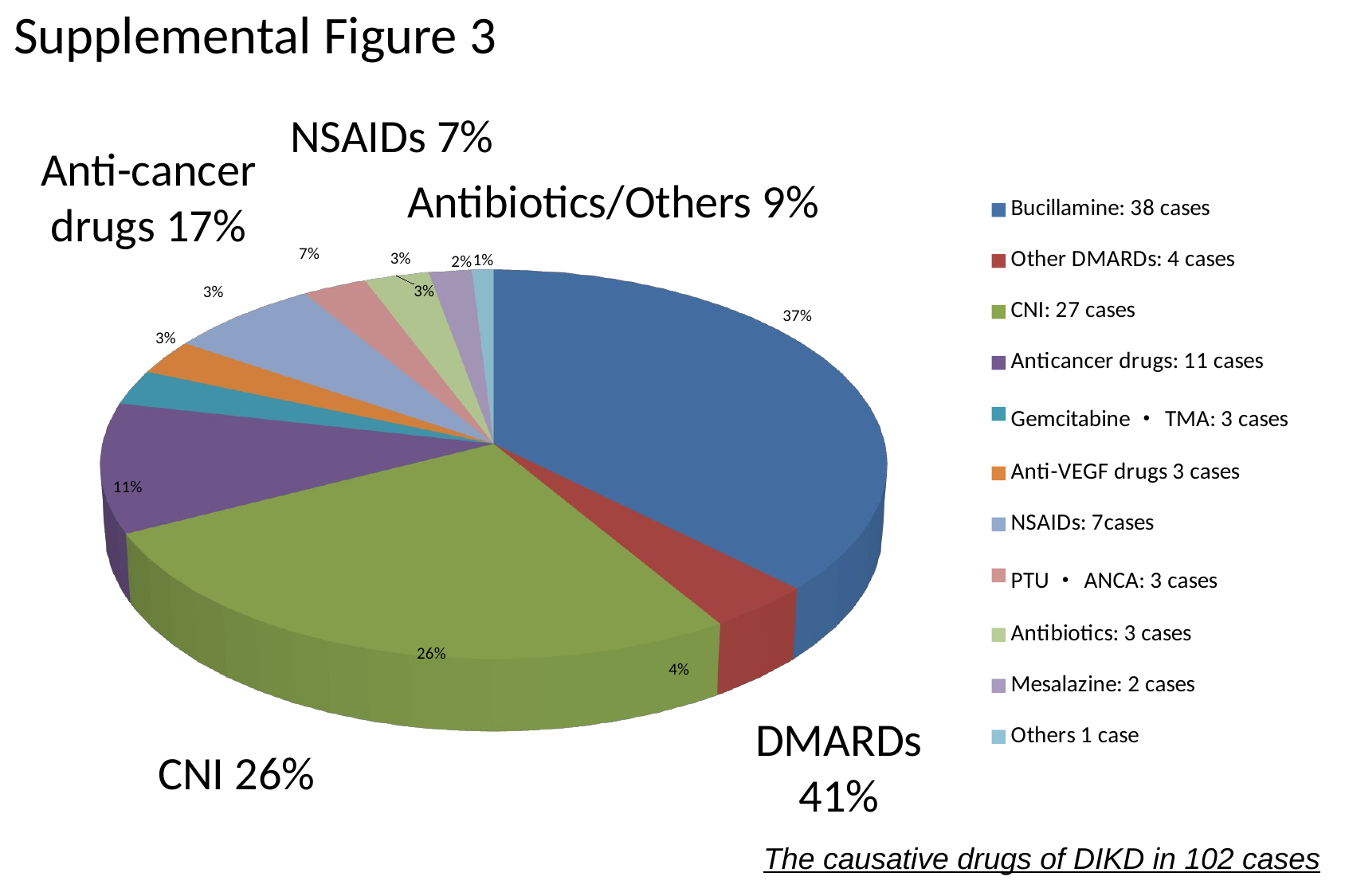

Supplemental Figure 3
NSAIDs 7%
Anti-cancer drugs 17%
[unsupported chart]
Antibiotics/Others 9%
DMARDs
41%
CNI 26%
The causative drugs of DIKD in 102 cases
